# Comparative Analysis of Perovskite Solar Cells for Obtaining a Higher Efficiency Using a Numerical Approach

**DOI:** 10.3390/mi14061127

**Published:** 2023-05-27

**Authors:** Khaled Hussein Mahmoud, Abdullah Saad Alsubaie, Abdul Hakeem Anwer, Mohd Zahid Ansari

**Affiliations:** 1 Department of Physics, College of Khurma University College, Taif University, P.O. Box 11099, Taif 21944, Saudi Arabia; k.hussein@tu.edu.sa (K.H.M.);; 2 School of Mechanical Engineering, Yeungnam University, Gyeongsan 38541, Republic of Korea; hakeemanwer1@gmail.com; 3 School of Materials Science and Engineering, Yeungnam University, 280 Daehak-Ro, Gyeongsan 38541, Republic of Korea

**Keywords:** PSCs, MASnI_3_, CsPbI_3_, PCE, computational and numerical modeling

## Abstract

Perovskite materials have gained considerable attention in recent years for their potential to improve the efficiency of solar cells. This study focuses on optimizing the efficiency of perovskite solar cells (PSCs) by investigating the thickness of the methylammonium-free absorber layer in the device structure. In the study we used a SCAPS-1D simulator to analyze the performance of MASnI_3_ and CsPbI_3_-based PSCs under AM1.5 illumination. The simulation involved using Spiro-OMeTAD as a hole transport layer (HTL) and ZnO as the electron transport layer (ETL) in the PSC structure. The results indicate that optimizing the thickness of the absorber layer can significantly increase the efficiency of PSCs. The precise bandgap values of the materials were set to 1.3 eV and 1.7 eV. In the study we also investigated the maximum thicknesses of the HTL, MASnI_3_, CsPbI_3_, and the ETL for the device structures, which were determined to be 100 nm, 600 nm, 800 nm, and 100 nm, respectively. The improvement techniques used in this study resulted in a high power-conversion efficiency (PCE) of 22.86% due to a higher value of V_OC_ for the CsPbI_3_-based PSC structure. The findings of this study demonstrate the potential of perovskite materials as absorber layers in solar cells. It also provides insights into improving the efficiency of PSCs, which is crucial for advancing the development of cost-effective and efficient solar energy systems. Overall, this study provides valuable information for the future development of more efficient solar cell technologies.

## 1. Introduction

In the recent decade, the perovskite solar cell has acquired a great sort of interest amongst scientists and researchers around the world. The typical perovskites, which are organometallic halides having the structural composition of ABX_3,_ have played a great role in transforming the hybrid-solar-cell field. Since the structural composition of ABX_3_ [1,2,3] includes A as methylammonium cation (MA^+^), which has a much larger ionic radius (i.e., 0.18 nm) than the other two ions (B and X), the other cation that is used, (i.e., B) is typically Pb or Sn or Ge, while the halide materials are I, Cl, Br, etc. [4,5]. In the past decade, perovskite solar cells (PSCs) have emerged as one of the best alternatives as they have exceptional optoelectronic properties, such as a visible solid absorption range, along with extended carrier diffusion lengths, and improved efficiency [6,7,8,9,10]. The semiconductor materials that make up solar cells allow them to produce energy directly from sunlight thanks to the photovoltaic effect. A solar cell absorbs less of the higher-energy photons than the bandgap when light strikes it. In 2009, Kojima et al. constructed CH_3_NH_3_PbX_3_-based PCE and reported up to 3.8% efficiency utilizing Br as well as I as the halogen materials [9,11]. Following this, research for improving the efficiency of PSC devices grew exponentially to date. In comparison, a superior PSC device can be achieved by selecting suitable materials at optimized thicknesses for simulated PSC structures. It is also mandatory to optimize the device to check the appropriate thickness since the best operating thickness can reduce the cost of the PSC during fabrication. In this context, Kim et al. enriched the efficiency of a PSC device to 6.5% using TiO_2_ as the ETL, and using 2,20,7,70-tetrakis-(N, N-di-p-methoxyphenylamine)-9,90-spiro-borene (Spiro-OMeTAD) as post-annealing treatment to achieve a hole transport layer (HTL) with superior performance [12]. At the same time Liu et al. also used the vapor deposition technique for obtaining an improved PCE with up to 15.4% efficiency by creating a layer configuration consisting of ITO/TiO_2_/CH_3_NH_3_PbI_3-x_Cl_x_/Spiro-OMeTAD/Ag [13]. The simple and easy fabrication made these types of perovskite solar cells with planar heterojunction architecture an effective substitute for the mesoscopic architecture. On the other hand, the major problem in PSC-device fabrication is obtaining the best optoelectronic parameters through thickness optimization of the light-harvesting layer, as this influences the device’s output. It is worth noting that using a lesser thickness of perovskite (PVK) in PSCs results in a lower photon absorbance, leading to a decrease in photocurrent density. In a previous study, ZnO and Spiro-OMeTAD were chosen for the electron transport layer (ETL) and hole transport layer (HTL), respectively, and we used the same materials in our simulations to study the PSC-device outcomes. We evaluated ZnO and Spiro-OMeTAD as superior carrier transport materials for PSCs and carefully analyzed the impact of total defect density on PSC performance [13,14]. We also used the same in our simulation to study the outputs of the PSC device. Here, we evaluated ZnO and Spiro-OMeTAD as the superior carrier transport material in PSC for further investigations. Additionally, we also carefully analyzed the influence of the total defect density on the output performances of the simulated PSCs. Recent research by Lin et al. has shown that improving the hole contact layer is crucial for achieving high efficiency in Si heterojunction solar cells [15]. Similarly, selecting an appropriate band alignment is crucial for designing PSCs. A thicker PVK layer can lead to extended recombination and reduced charge extraction, making it challenging to achieve optimal PSC performance. Therefore, selecting an appropriate thickness with optimal donor and acceptor doping densities for all component layers in PSCs is the best approach to attain higher device outputs.

Designing perovskite-solar-cell devices requires a significant focus on selecting an appropriate band alignment in the PSC. Another crucial aspect to consider is the impact of the thickness of the PVK layer on charge extraction, as a thicker PVK layer can lead to increased recombination, which poses a more significant challenge for PSC devices. Previous studies have reported that the PVK layer typically ranges from 0.1 µm to 0.35 µm [16]. Lead-based materials, particularly methylammonium lead iodide (MAPbI_3_), have been the focus of significant research efforts aimed at enhancing the performance of perovskite solar cells in recent years. However, the toxic nature of lead has limited its widespread use. As a solution, tin (Sn) can be used as a replacement for lead (Pb) in the absorber layer. Previous studies have demonstrated that MASnI_3_ has a band gap of approximately 1.3 eV, which is lower than that of CsPbI_3_ (~1.7 eV). As a result, MASnI_3_ is a promising alternative candidate for use in PSCs [7,17,18]. MASnI_3_ has a smaller bandgap, which allows it to absorb a wider range of incident photon wavelengths compared to CsPbI_3_, but the latter exhibits a higher value of V_OC_, which contributes to higher PSC-device outputs. Despite the fact that the MA cation produces the highest experimental efficiency, its volatile nature can negatively impact the effectiveness of MA-based perovskite solar cells. Additionally, the effective fabrication of the MA cation is not very stable due to its organic nature. Due to these concerns, we decided to exclude the MA cation and instead used the Cs cation. In recent times, the efficiency of Cs-based perovskite has greatly improved compared to MA, so we also included Cs-based perovskite in our solar cell simulation. As a result, we chose CsPbI_3_ as the absorber layer for our study because MASnI_3_ is thermally unstable due to the presence of the methylammonium component in the metal compound in PSC devices. This allowed us to evaluate their efficiency and other optoelectronic parameters [19,20].

In addition to experimental work, theoretical modeling techniques are used to study solar cell parameters and optimize PSCs. These modeling techniques are essential for designing solar cells because they provide a more cost- and time-effective way of testing and evaluating various design options. Without numerical techniques, designing a solar cell would be unreliable and costly, as a large number of trial-and-error experiments would be required to arrive at the optimal design. Therefore, theoretical modeling techniques play a crucial role in the development of efficient and cost-effective PSCs [21]. The use of theoretical modeling techniques not only reduces the risk involved in designing PSCs but also allows for a thorough investigation of the characteristics of the constituent layers. This approach provides a more detailed understanding of the factors that affect a solar cell’s performance and enables the optimization of various performance parameters. Therefore, the strategy of using theoretical modeling techniques offers a more comprehensive and efficient approach to designing PSCs [22]. Furthermore, using real-world examples of PSCs, the application of numerical simulation techniques provides a practical approach to evaluating the significance of numerous material properties. This approach enables the evaluation of various design options and material combinations before investing significant time and resources in experimental testing. Moreover, numerical simulations have become particularly critical in the field of material science as it advances and seeks to discover novel functional materials. These simulations provide invaluable insights into the known characteristics of materials and can guide future research by predicting the fastest and most effective method for discovering new materials. Therefore, numerical simulations play a crucial role in both designing efficient PSCs and advancing the field of material science.

In this study, we utilized a solar illumination of 1.5AM to evaluate the optical profile of the perovskite solar cell (PSC) structure. Using simulation software, we investigated the carrier transport phenomena and electric outputs at different vacuum level bandgaps for PSCs based on both MASnI_3_ and CsPbI_3_ materials. Additionally, we examined the capacitance–voltage parameters (C–V) for the optimized PSC device. The primary parameters of the PSCs, including short circuit current density (J_sc_), open-circuit voltage (V_oc_), fill factor (FF), and power-conversion efficiency (PCE), were explored for the optimized thickness of each constituent layer in the MASnI_3_- and CsPbI_3_-based PSC structures. By analyzing these parameters, we aim to identify the most efficient and effective PSC design for practical applications.

## 2. Simulated Device Structure

**Figure 1:**
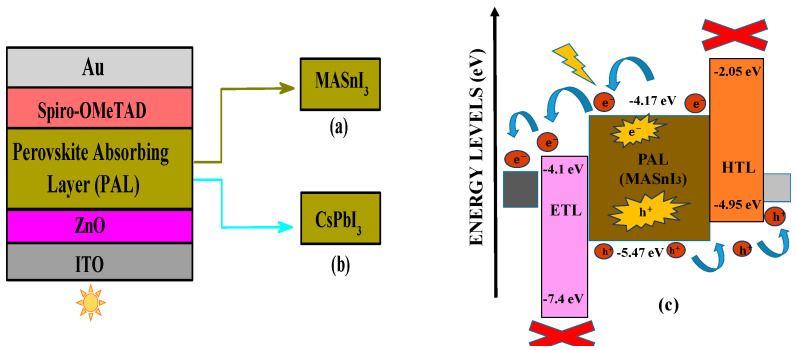
Representations of the layers for both the PSC devices, i.e., (**a**) D1, (**b**) D2, and (**c**) energy-level diagram for the PSC devices at the illuminance of AM1.5.

An n-i-p configured PSC with D1 (ITO/ZnO/MASnI_3_/Spiro-OMeTAD/Au) and D2 (ITO/ZnO/CsPbI_3_/Spiro-OMeTAD/Au) is utilized in the extended numerical simulation in the PSC as depicted in Figure 1a,b. The D2 device is considered specifically due to the volatile nature of MA cation in the PSC architecture as the archetypical PSC is built with ITO as the front electrode with a thickness level of 100 nm. Along with that, 100 nm thin ZnO is used which functions as the ETLs, as it effectively gathers the generated electrons from absorber layers in the simulated PSCs. For both the devices (D1 and D2), the PVK layer has a precise thickness of 200 nm as it is sandwiched between both the carrier transport layers (CTL). Due to the thickness of 100 nm of the HTL (Spiro-OMeTAD) efficiently assembles the hole from the absorber and immediately transmits it to the back contact with the drift-diffusion mechanism. Here, the back contact is Au, which has a thickness level of 100 nm. The input parameters were chosen based on previously published work. The drift-diffusion mechanism is shown in Figure 1c with the proper function of the electron–hole generation and transportation mechanism. Whenever the photons touch the active layer, the perovskite absorber generates excitons, which split into both electrons and holes. The generated electrons and holes transmit through the ETL and HTL, respectively, after which both the carriers are collected by the front and back contacts, as shown in Figure 1c.

In the present work, we carried out a widespread exploration of PSC devices utilizing a one-dimensional solar cell capacitance simulator, which is popularly known as SCAPS-1D, which was developed by ELIS, University of Ghent, Ghent, Belgium. The basic work principle of the simulator is the drift-diffusion mechanism [23]. The SCAPS-1D software has some exclusive features, such as the ability to deposit up to seven layers of semiconductor and to stimulate cells in both light and dark situations by solving the continuity and Poisson equations. It also incorporates progressive models, such as the interface-defect-level mechanism and recombination, to investigate trap losses in the PSCs [24]. Additionally, the optimization technique can be performed in batches using different parameters [25]. The recorded setup is considered one of the most important and superior optimization techniques available in the SCAPS-1D software. The SCAPS-1D simulator not only aids in effective design but also saves time and manufacturing costs in experimental design. This can lead to more efficient PSC design in the same operating environment as other, more expensive, simulators.

## 3. Computational Modeling

The computational simulation approach is used to explain and simplify the fundamental phenomena in solar devices and it reveals how basic factors impact optimal device output. It is important to note that without numerical modeling, building a solar cell would be impractical due to increased costs and longer lead times [13,21]. The technique eliminates the necessity for potentially dangerous examinations of the properties of component layers and offers a comprehensive analysis of optimizing solar cell performance. In addition, the method is a useful way to weigh the significance of different material qualities using real-world examples from solar cells. Numerical simulation has grown in importance, especially in the area of material science [26], making wise recommendations on the well-known characteristics of materials. It should be mentioned that the significant parameters impacting the device output of the PSC can be achieved by expanding the simulation modeling of SCAPS-1D, which was developed by Burgelman et al. [27]. The 1-D equation uses the steady-state diffusion condition of the semiconductor materials, and the correlation between the electric fields (E) and the charge density of the p–n junction can be characterized as given below [28,29]:

(1) $$\frac{\partial^{2}\varphi }{\partial^{2}x}=-\frac{\partial E}{\partial x}=-\frac{\rho }{\varepsilon_{s}}=-\frac{q}{\varepsilon_{s}}\left[p-n+N_{D}^{+}(x)-N_{A}^{-}(x)\pm N_{def}\left(x\right)\right]$$

Here, φ is the electrostatic potential, whereas q is the charge; $\varepsilon_{s}$ is called the medium’s static relative permittivity; *n* and p are the electrons and the hole, respectively, while $N_{A}^{+}$ and $N_{D}^{+}$ are the density of the acceptor and donor, respectively; and $N_{def}\left(x\right)$ is the defect density of both the acceptor and donor.

The carrier continuity equation in the PSC structure can be characterized as follows, where, $j_{p}$ and $j_{n}$ are the hole and electron current density, G is the carrier generation rate, and $U_{n}\left(n,p\right)$ and $U_{p}\left(n,p\right)$ are the recombination rates of electron and hole, respectively [25].

(2) $$-\frac{{\partial j}_{p}}{\partial x}+G-U_{p}\left(n,p\right)=0$$

(3) $$\frac{{\partial j}_{n}}{\partial x}+G-U_{n}\left(n,p\right)=0$$

The current density of both the carriers is obtained from the equation given below,

(4) $$j_{p}=qn\mu_{p}E-qD_{p}\frac{\partial p}{\partial x}$$

(5) $$j_{n}=qn\mu_{n}E+qD_{n}\frac{\partial n}{\partial x}$$

where q is the charge,$\mu_{p}$ and $\mu_{n}$ are the carrier mobilities, and $D_{p}$ and $D_{n}$ are the diffusion coefficients of the carriers.

The SCAPS-1D simulator usually extracts all the solar cell’s fundamental equations, such as the recombination rate, generation, and current density as well.

## 4. Results and Discussions

This section of the study is divided into seven parts: perovskite thickness optimization, temperature optimization, defectivity optimization, capacitance–impedance analysis, quantum efficiency (QE) analysis, and J–V analysis of both devices. All the simulations in this section were conducted with a standard illumination condition of AM1.5 and a power input of 1000 W/m^2^, using the predictable solar spectrum at ϴ_z_ = 48.20. This is because the majority of the world’s population lives in regions with moderate weather conditions, between the Arctic and the tropics. Hence, the results of the simulations are considered under the standard spectrum of AM1.5 for solar cell architecture, with an intensity of radiation of 1000 W/m^2^.

### 4.1. Comparison of Perovskite Thicknesses of Both the PSC Devices

**Figure 2:**
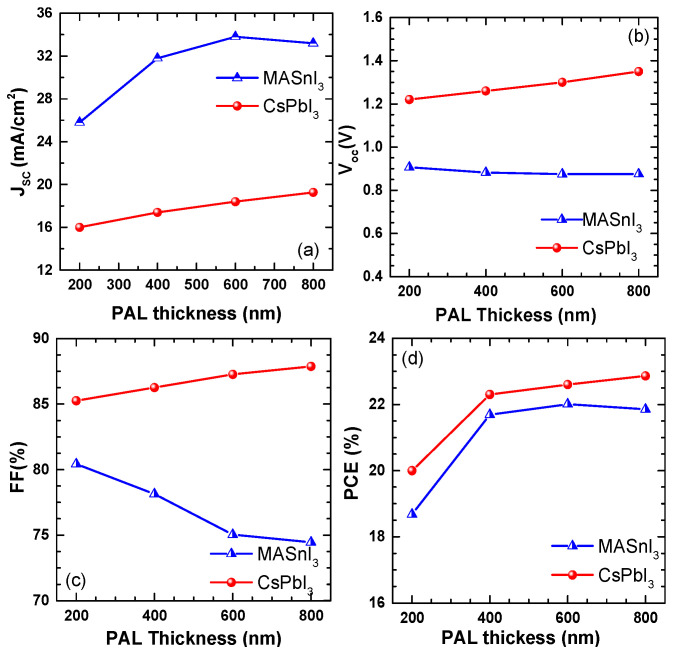
Perovskite-solar-cell outputs over different thicknesses of active layers. (**a**) short circuit current density (J_sc_); (**b**) open-circuit voltage V_oc_; (**c**) fill factor (FF); (**d**) power-conversion efficiency (PCE).

In this study we conducted an extensive analysis to determine the optimal operating temperature for the selected parameters. The results of this analysis are listed in Table 1, with all simulations conducted at a working temperature of 300 K [26,27]. Figure 2 illustrates the impact of varying the absorber-layer thickness on four PV parameters of the simulated PSC devices D1 and D2. The results were obtained after validation and analysis of D1 and D2. It can be observed in Figure 2a that the short circuit current density (J_sc_) increased for both devices as the absorber layer thickness increased. For D1, J_sc_ reached a saturated value of 19.29 mA/cm^2^ at 800 nm; for D2, J_sc_ reached a saturated value of 33.62 mA/cm^2^ at 600 nm. This study highlights that the thickness of the PVK layer is critical in enhancing the exciton-generation rate, which leads to a higher absorbance in the smaller bandgap of absorber layers. Therefore, it is suggested that the optimal thickness for the PVK layer should be between 600 nm and 800 nm for both PSC devices (D1 and D2) with bandgaps of 1.3 eV and 1.7 eV, respectively. This increase in the exciton-generation rate can effectively increase the current in the active layer of both PSC devices.

Figure 2b shows the effect of PVK layer thickness on the V_oc_ values for thicknesses ranging from 200 nm to 800 nm. As illustrated in the figure, the V_oc_ values increased with the increasing thickness of the PVK layer. This can be attributed to the higher absorption of photons by the absorber layer, resulting in an increased exciton-generation rate and a greater number of electron–hole pairs generated. This led to a higher built-in potential and an increase in the V_oc_ value. Interestingly, the V_oc_ for D1 showed a non-monotonic change up to 400 nm thickness, where most of the photons were absorbed by this precise thickness in the active layer. On the other hand, V_oc_ for D2 showed a monotonic dependency on the thickness variation. These observations suggest that the optimal thickness of the PVK layer may vary depending on the bandgap of the absorber layer and the device architecture. Further studies are needed to investigate the underlying mechanisms behind these observations and to optimize the device performance accordingly. Overall, these results highlight the importance of optimizing the thickness of the PVK layer in order to achieve higher efficiency in PSC devices. However, the FF decreased steeply in both PSC devices when the thickness of the absorber increased from 200 nm to 800 nm, whereas the D2 material slightly improved for the D2 device, as depicted in Figure 2c. The variation in the PCE for both PSCs over the active layer thickness is presented in Figure 2d. As can be seen, D1 reached a PCE of 22.01%, and D2 reached 22.86% at a thickness of 600 nm and 800 nm, respectively. Both devices attained significantly higher solar cell outputs compared to Bhattarai et al. and Hossain et al. [25,26]. The considerable increase in V_OC_ for both active layer thicknesses of 600 nm and 800 nm was due to the higher generation of exciton pairs, which significantly affected the PCE of the PSC devices. The study of thickness variation not only leads to cost savings in the production process but also streamlines the manufacturing process of PSC devices.

### 4.2. Comparison of Efficiency at Different Working Temperatures and Series Resistances for Both the PSC Devices

Optimizing the working temperature is an important factor in the development of perovskite solar cells. PSCs are expected to operate at higher temperatures compared to traditional solar cells, which can lead to thermal instability and reduced performance. Therefore, researchers are exploring various approaches to improve the thermal stability of PSCs and identify the most suitable working temperature for optimal performance. Some of the techniques being investigated include modifying the composition of the perovskite layer, incorporating new materials into the device structure, and optimizing the fabrication process to reduce defects and enhance stability. As solar cells typically operate at temperatures between 300 K and 400 K, the recently developed PSCs must be able to withstand higher temperatures. This thermal instability presents a significant challenge for PSCs, and researchers are actively working to find ways to determine the most suitable temperature for studying PSC devices. The PSCs are expected to operate at higher temperatures to achieve the higher efficiency of the PSCs compared to conventional solar cells. Definitely, traditional solar cells can also operate at higher temperatures, but the effectivity of PSCs is greater than that of the conventional cells. Such devices retain over 80% of their initial power conversion efficiency (PCE) after heating at 200 °C for 45 h, enabling their operation at high temperatures as confirmed by Dong et al. [28].

**Figure 3:**
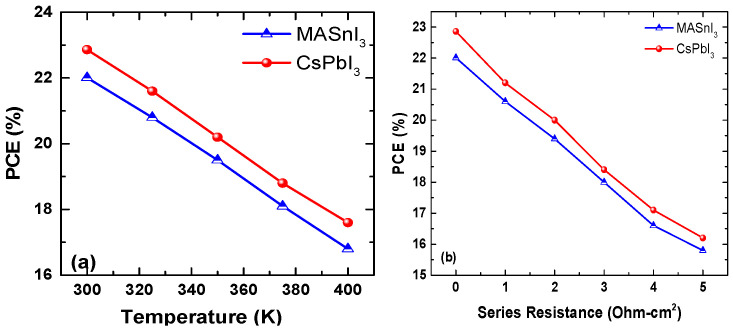
Perovskite-solar-cell outputs over different temperatures (**a**) and series resistances (**b**).

Figure 3a shows that both simulated devices’ power conversion efficiency (PCE) decreased as the temperature increased from 300 K to 400 K. The decrease in PCE was more significant at higher temperatures, indicating that the impact of higher temperatures on both devices is damaging. This decreasing trend in PCE with temperature was consistent across different temperatures, as shown in the exploration of five different temperatures. It is known that the interior structures of the perovskite deform at higher temperatures. The PSCs reached a PCE of 22.01% for D1 and 22.86% for D2 at a temperature of 300 K. At a higher temperature, 400 K, they reached the lowest PCEs of 16.2% and 17.8%. Figure 3b illustrates that increasing the series resistances resulted in a smaller value of PCE. The study shows that the smallest value of 15.8% and 16.2% was reached at the upper value of series resistances of up to 5 Ohm-cm^2^, as displayed in Figure 3. This result is consistent with a previous study by Bhattarai et al. [17]. The overall outputs of the PSC also decreased with the increase in temperature and series resistance.

### 4.3. Comparison of Efficiency at Different Defect Densities for Both the PSC Devices

Defect densities are another crucial parameter that significantly affected the overall output of the PSCs in the current simulation. The performance of PSCs is greatly influenced by the total defect density, which is caused by incomplete dangling bonds in the material and by trap energy levels within the bandgap that serve as recombination centers, and which shortens carrier lifetimes. Passivating these defect levels through chemical, compositional, or material engineering techniques can reduce the defect density and extend carrier lifetimes, leading to improved PSC performances. Therefore, controlling the defect density is critical for enhancing the efficiency and stability of PSCs.

**Figure 4:**
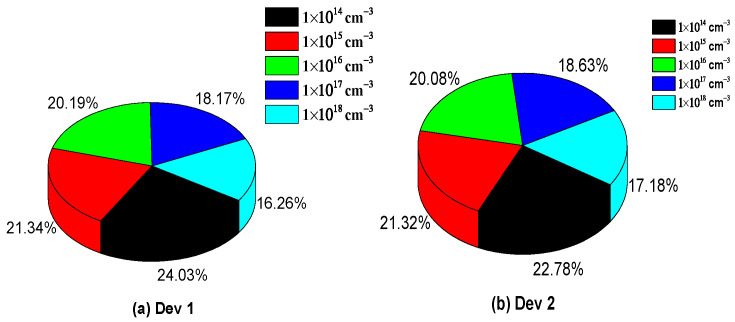
(**a**,**b**) Perovskite-solar-cell outputs over the different defect densities of active layers for both PSC devices.

For these reasons, the defect density analysis was used in the present study, as this method helps improving the efficiency of PSC devices. The defectivity of the simulated PSCs studied ranged from 1 × 10^14^ cm^−3^ to 1 × 10^18^ cm^−3^, as shown in Figure 4. At first, D1 offered a high efficiency of 22.01%, whereas it kept decreasing down to 14.4% when the total defect density increased to 1 × 10^18^ cm^−3^, as shown in Figure 4a. A similar trend was attained for D2 which, at a level of total defect density of 1 × 10^14^ cm^−3^, obtained the superior value of 22.86%, while 17.1% efficiency was attained for the active layers with a higher defect density, 1 × 10^18^ cm^−3^, as shown in Figure 4b. The pie chart describes which efficiency fractions were obtained for both PSC devices. The change in fractions with the defects can be obtained from the pie chart, as depicted in Figure 4 [29].

Furthermore, in Table 2, the numerical values at a defect density of 1 × 10^14^ cm^−3^ are provided for the overall outputs of the simulated solar cell. The presence of incomplete dangling bonds during the fabrication process leads to additional defect levels within the material’s bandgap. These defect levels act as recombination centers and affect the carrier lifetime and diffusion length. To check these defect-related effects, the total defect density within the material was considered in the simulation to obtain more realistic results for the device’s performance.

### 4.4. Capacitance Analysis in the Optimized CsPbI_3_-Based PSC Device

**Figure 5:**
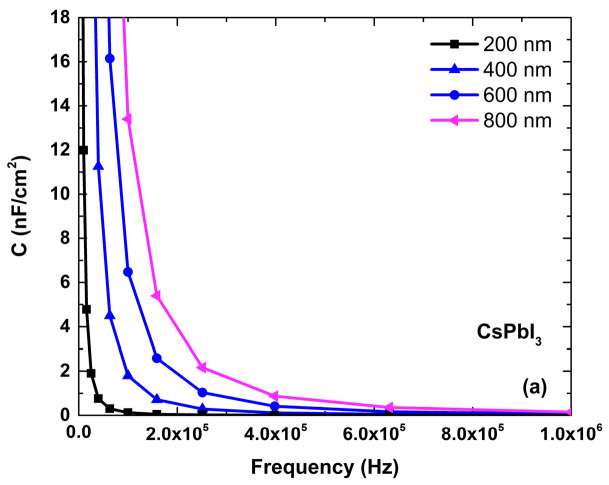

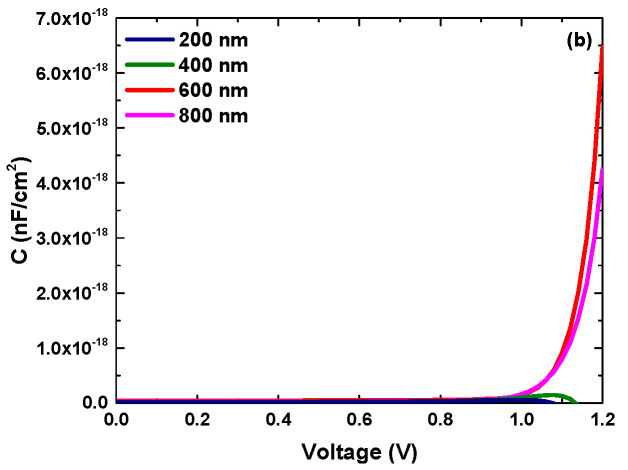
(**a**) Capacitance of the perovskite-solar-cell device structure at different frequencies and (**b**) different voltages.

Figure 5a shows the capacitance values for the optimized PSC device at different frequencies and thicknesses. The cutoff value increased as the thickness increased. In Figure 5b, the maximum capacitance value is observed at higher thicknesses, reaching a value of 6 × 10^18^ nF-cm^−2^ at a thickness of 800 nm. The value shows that the simulated CsPbI_3_-based PSC device showed better results for 800 nm thickness than other layer thicknesses of the CsPbI_3_ absorber. On the other hand, the C–V curve at a simulated frequency value of 10^6^ Hz, shows the impact of frequency on the capacitance–voltage curve for the CsPbI_3_-based PSC device.

### 4.5. Impedance Analysis for the CsPbI_3_-Based PSC Device

**Figure 6:**
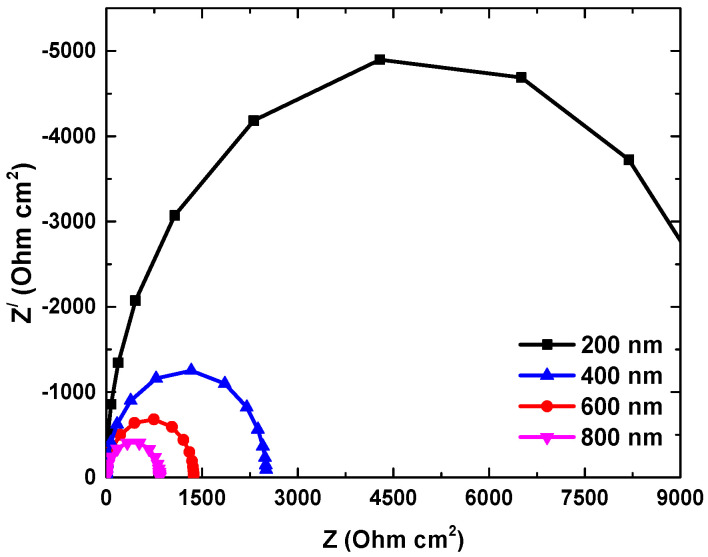
Impedance of the optimized perovskite solar cell at different thicknesses.

The impedance analysis of the present work emphasized that a minimum impedance value can be seen, as shown in Figure 6 below. The Nyquist plot or (-Im Z- Real Z) was determined using an extra RC component in the PSC device circuits. As we further increased the thickness, the value of the impedance became smaller. For comparison, the 200 nm thickness of the perovskite layer offered a value of nearly 5000 Ohm-cm^2^. However, the 800 nm thickness obtained a value of 250 Ohm-cm^2^. The size of the Nyquist semicircle in the impedance plot can be influenced by different thickness levels of the perovskite absorber layer. Generally, a larger Nyquist semicircle indicates a higher charge transfer resistance at the absorber/transport layer (CTL) interface. An earlier study by Bhattarai et al. also showed a smaller impedance value for the perovskite-solar-cell device [17].

### 4.6. Quantum Efficiency (QE) Parameters for Optimized PSC Devices

It should be mentioned that the quantum efficiency (QE) of a PSC primarily depends on the absorption properties of the absorber layer. However, other layers in the device can also contribute to the overall QE. In a typical thin-film solar cell architecture, the absorber layer is situated between the transparent conductive oxide (TCO) layer and the cathode layer. The TCO layer is responsible for transmitting incident light into the absorber layer, while also serving as the front contact for the device. The back contact layer provides a pathway for charge extraction. The TCO and back contact layers are critical components of the photovoltaic device, and their impact on the QE is significant. The TCO layer plays a crucial role in determining the amount of light that reaches the absorber layer. If the TCO layer is too thick or too resistive, it can limit the amount of light that enters the absorber layer, resulting in a reduction in the QE. Similarly, the back contact layer can influence the QE by affecting the device’s charge extraction efficiency. In addition, other layers, such as buffer layers, interfacial layers, or charge transport layers, may be incorporated into the solar cell structure to enhance the device’s performance. The choice of material and thickness of these layers is critical in achieving high device efficiency. Therefore, optimizing the solar cell structure involves a careful balance of all the layers to achieve high QE and device performance. For example, in perovskite solar cells, an electron transport layer and a hole transport layer are often included to improve charge transport and reduce recombination losses. These layers can also affect the QE by influencing the efficiency of charge extraction and recombination within the device. In summary, while the absorber layer is the primary contributor to the QE in a solar cell, other layers in the device can also have an impact on the overall QE. The performance of a solar cell is the result of a complex interplay between the various layers and their properties, and optimizing each layer is critical to achieving high device efficiency.

**Figure 7:**
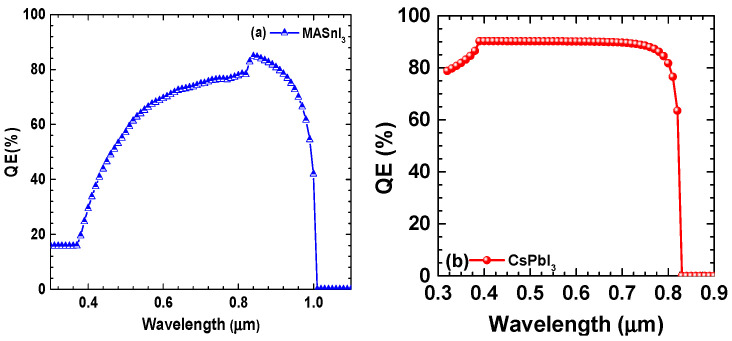
(**a**,**b**) The quantum efficiency (QE) over the function of the wavelength for two different materials.

Figure 7 depicts the QE parameter under the incident wavelength of photons for designed CsPbI_3_-based PSCs. It can be noted that the QE of the PSC is inversely proportional to the bandgap of the PVK layer. The D1 device with MASnI_3_ shows a smaller fraction of QE; i.e., only 70% in the entire wavelength ranging from 300 to 1100 µm as shown in Figure 7a,b. The CsPbI_3_ has a higher bandgap (1.67 eV) compared to the MASnI_3_, which is why the smaller high range of wavelength absorption up to 0.85 µm is possible for the latter one. While high EQE can be observed for CsPbI_3_, due to the higher bandgap, more energy is required to excite electrons from the valence band compared to MASnI_3_-based PSCs, as shown in Figure 7. For comparison, the present simulation of the CsPbI_3-_based PSC offers a much higher QE value than the value previously reported by Bhattarai et al., which was 85% on average [17].

### 4.7. Comparison of J–V Parameters for Both the Devices

**Figure 8:**
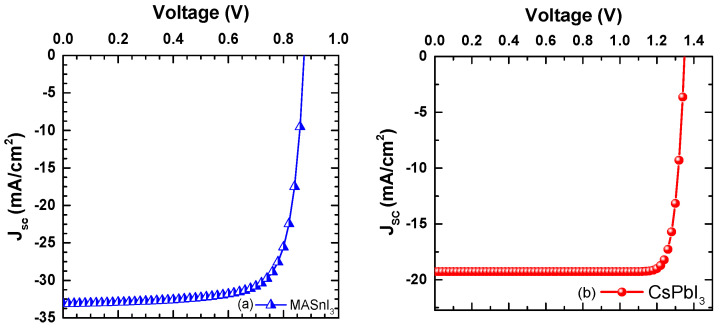
(**a**,**b**) J–V of two different perovskite materials at the optimized conditions.

Figure 8 compares the current density over a biased voltage curve (J–V) function for D1 and D2 devices under AM1.5 solar illumination. It can be seen that the current density for both devices, i.e., D1 and D2, remained steady with rising voltage to 0.7 V and 1.2 V, then dropped suddenly afterwards with further increasing voltage. This is due to the more extensive absorption by the insertion of both the perovskite materials. For the D1 device, an improved V_OC_ of 0.875 V was observed, whereas D2 obtained a higher value of V_OC_ of 1.35 V. Apart from that, the higher value of PCE was also due to the contribution of FF as the square or rectangle that we can fit on the J–V curve shows the FF parameter. So, due to the steeper value of the J–V curve, we obtained a higher value of FF for CsPbI_3_ compared to the MASnI_3_-based PSC device. The higher bandgap of CsPbI_3_ led to a higher V_OC_ value, ultimately leading to obtaining a higher value of PCE, as shown in Table 3. The present work shows the same trend as studies by Bhattarai et al. [17] and Hima et al. [30]. Particularly, the extensive absorption property in the active layer of the smaller bandgap PSC resulted in the larger short circuit current density that further led to the formation of higher rates of electron and hole pairs under AM1.5 solar irradiation. After the maximum optimization, the CsPbI_3_-based PSC offered an efficiency of up to 22.86%, which can provide an effective route for further optimization of lead-free, Cs-based PSC devices.

## 5. Conclusions

In this study, we investigated the carrier transport phenomena for both lead-based and lead-free perovskite solar cells (PSCs) under AM1.5 illumination. Our results showed that the absorber layer plays a significant role in determining the electrical parameters of PSCs. We optimized the PSC parameters for both lead-based and lead-free devices and obtained the highest power conversion efficiency (PCE) for a methylammonium-free PSC device with a J_sc_ of 19.29 mA/cm^2^, V_oc_ of 1.35 Volt, FF of nearly 87.86%, and PCE approaching nearly 22.86%. Moreover, the simulated data for both device structures showed an improvement in PCE compared to previously reported values. We also performed capacitance vs. voltage (C–V) analysis to ensure the reliability of the PSCs. Our study offers recommendations for the fabrication of lead-free CsPbI_3_-based PSC devices and identifies additional work that can be performed to further improve their efficiency. Furthermore, our study addresses the concerns of thermal instability in perovskite films due to annealing, which is a crucial step in their fabrication. We found that the PCE of both lead-based and lead-free PSCs decreases with increasing temperature, indicating the need for PSCs that can withstand high temperatures. Overall, our study provides valuable insights into the carrier transport phenomena in PSCs and offers recommendations for their fabrication and improvement.

## Figures and Tables

**Table 1:** The chosen inputs in simulating the PSC devices [6,17,24].

Parameter	Terms	ETL (ZnO)	PVK_1_ (MASnI_3_)	PVK_2_ (CsPbI_3_)	HTL (Spiro-OMeTAD)
d (nm)	Thickness	100	600	800	100
E_g_ (eV)	Bandgap	3.3	1.3	1.694	2.9
*χ*	Electron affinity	4	4.1	3.95	2.45
*ε*_r_	Relative permittivity	9	8.2	6	3
N_c_ (cm^−3^)	Effective DoS at CB	3.7 × 10^18^	2 × 10^18^	1.1 × 10^20^	1 × 10^19^
N_v_ (cm^−3^)	Effective DoS at VB	1.8 × 10^19^	2 × 10^18^	8.2 × 10^30^	1 × 10^19^
µ_n_ (cm^2^/Vs)	Mob. of electrons	100	1.6	25	2 × 10^−4^
µ_p_ (cm^2^/Vs)	Mob. of holes	25	1.6	25	2 × 10^−4^
D_a_ (cm^−3^)	Dop. conc. of the acceptor	0	0	1 × 10^15^	2 × 10^18^
D_d_ (cm^−3^)	Dop. conc. of donor	1 × 10^18^	1 × 10^19^	0	0
Nt (cm^−3^)	Def. density	1 × 10^14^	1 × 10^14^	1 × 10^14^	1 × 10^14^

**Table 2:** Interface-defect-density details.

Interface	Defect Type	Cap. Cross Section: Electrons/Holes (cm^2^)	Energetic Distribution	Ref. for Defect Energy Level	Total Density (cm^−2^)
ETL/PAL	Neutral	1.0 × 10^−19^ 1.0 × 10^−19^	Single	Above the VB maximum	1.0 × 10^10^
PAL/HTL	Neutral	1.0 × 10^−19^ 1.0 × 10^−19^	Single	Above the VB maximum	1.0 × 10^10^

**Table 3:** Simulated PV device characteristics along with earlier works.

Device Configuration	J_sc_ (mA/cm^2^)	V_oc_ (V)	FF (%)	PCE (%)
MASnI_3_-based PSC	33.62	0.875	75.94	22.01
CsPbI_3_-based PSC	19.29	1.35	87.86	22.86 (23%)
Bhattarai et al. [17]	33.19	0.876	76.19	22.16
Hima et al. [30]	18.63	0.8	64.8	9.56

## References

[1] Raoui Y., Ez-Zahraouy H., Kazim S., Ahmad S. (2021). Energy level engineering of charge selective contact and halide perovskite by modulating band offset: Mechanistic insights. J. Energy Chem..

[2] Bhattarai S., Mhamdi A., Hossain I., Raoui Y., Pandey R., Madan J., Bouazizi A., Maiti M., Gogoi D., Sharma A. (2022). A detailed review of perovskite solar cells: Introduction, working principle, modelling, fabrication techniques, future challenges. Micro Nanostruct..

[3] Snaith H.J. (2013). Perovskites: The Emergence of a New Era for Low-Cost, High-Efficiency Solar Cells. J. Phys. Chem. Lett..

[4] Teimouri R., Mohammadpour R.J.S. (2018). Potential application of CuSbS2 as the hole transport material in perovskite solar cell: A simulation study. Superlattices Microstruct..

[5] Supasai T., Rujisamphan N., Ullrich K., Chemseddine A., Dittrich T. (2013). Formation of a passivating CH3NH3PbI3/PbI2 interface during moderate heating of CH3NH3PbI3 layers. Appl. Phys. Lett..

[6] Bhattarai S., Das T.D. (2021). Optimization of carrier transport materials for the performance enhancement of the MAGeI3 based perovskite solar cell. Sol. Energy.

[7] Bhattarai S., Hossain I., Maiti M., Pandey R., Madan J. (2023). Performance analysis and optimization of all-inorganic CsPbI3-based perovskite solar cell. Indian J. Phys..

[8] Bhattarai S., Pandey R., Madan J., Muchahary D., Gogoi D. (2022). A novel graded approach for improving the efficiency of Lead-Free perovskite solar cells. Sol. Energy.

[9] Bhattarai S., Sharma A., Das T.D. (2020). Efficiency enhancement of perovskite solar cell by using doubly carrier transport layers with a distinct bandgap of MAPbI3 active layer. Optik.

[10] Park N.-G. (2015). Perovskite solar cells: An emerging photovoltaic technology. Mater. Today.

[11] Bhattarai S., Sharma A., Muchahary D., Gogoi D., Das T.D. (2021). Numerical simulation study for efficiency enhancement of doubly graded perovskite solar cell. Opt. Mater..

[12] Kim H.-S., Lee C.-R., Im J.-H., Lee K.-B., Moehl T., Marchioro A., Moon S.-J., Humphry-Baker R., Yum J.-H., Moser J.E. (2012). Lead Iodide Perovskite Sensitized All-Solid-State Submicron Thin Film Mesoscopic Solar Cell with Efficiency Exceeding 9%. Sci. Rep..

[13] Liu D., Kelly T.L. (2014). Perovskite solar cells with a planar heterojunction structure prepared using room-temperature solution processing techniques. Nat. Photonics.

[14] Manabeng M., Mwankemwa B.S., Ocaya R.O., Motaung T.E., Malevu T.D. (2022). A Review of the Impact of Zinc Oxide Nanostructure Morphology on Perovskite Solar Cell Performance. Processes.

[15] Lin H., Yang M., Ru X., Wang G., Yin S., Peng F., Hong C., Qu M., Lu J., Fang L. (2023). Silicon heterojunction solar cells with up to 26.81% efficiency achieved by electrically optimized nanocrystalline-silicon hole contact layers. Nat. Energy.

[16] Stranks Samuel D., Eperon Giles E., Grancini G., Menelaou C., Alcocer Marcelo J.P., Leijtens T., Herz Laura M., Petrozza A., Snaith Henry J. (2013). Electron-Hole Diffusion Lengths Exceeding 1 Micrometer in an Organometal Trihalide Perovskite Absorber. Science.

[17] Bhattarai S., Pandey R., Madan J., Mhamdi A., Bouazizi A., Muchahary D., Gogoi D., Sharma A., Das T.D. (2022). Investigation of Carrier Transport Materials for Performance Assessment of Lead-Free Perovskite Solar Cells. IEEE Trans. Electron. Devices.

[18] Bhattarai S., Sharma A., Swain P.K., Das T.D. (2021). Numerical Simulation to Design an Efficient Perovskite Solar Cell Through Triple-Graded Approach. J. Electron. Mater..

[19] Hossain M.K., Toki G.F.I., Kuddus A., Rubel M.H.K., Hossain M.M., Bencherif H., Rahman M.F., Islam M.R., Mushtaq M. (2023). An extensive study on multiple ETL and HTL layers to design and simulation of high-performance lead-free CsSnCl3-based perovskite solar cells. Sci. Rep..

[20] Hossain M.K., Samajdar D.P., Das R.C., Arnab A.A., Rahman M.F., Rubel M.H.K., Islam M.R., Bencherif H., Pandey R., Madan J. (2023). Design and Simulation of Cs2BiAgI6 Double Perovskite Solar Cells with Different Electron Transport Layers for Efficiency Enhancement. Energy Fuels.

[21] Ebner M., Marone F., Stampanoni M., Wood V.J.S. (2013). Visualization and quantification of electrochemical and mechanical degradation in Li ion batteries. Science.

[22] Minemoto T., Murata M. (2014). Device modeling of perovskite solar cells based on structural similarity with thin film inorganic semiconductor solar cells. J. Appl. Phys..

[23] Burgelman M., Marlein J. Analysis of graded band gap solar cells with SCAPS. Proceedings of the 23rd European Photovoltaic Conference.

[24] Jayan K.D., Sebastian V., Kurian J. (2021). Simulation and optimization studies on CsPbI3 based inorganic perovskite solar cells. Sol. Energy.

[25] Bhattarai S., Sharma A., Das T.D. (2020). Factor affecting the performance of perovskite solar cell for distinct MAPI layer thickness. AIP Conf. Proc..

[26] Hossain M.K., Mohammed M.K.A., Pandey R., Arnab A.A., Rubel M.H.K., Hossain K.M., Ali M.H., Rahman M.F., Bencherif H., Madan J. (2023). Numerical Analysis in DFT and SCAPS-1D on the Influence of Different Charge Transport Layers of CsPbBr3 Perovskite Solar Cells. Energy Fuels.

[27] Bhattarai S., Pandey R., Madan J., Sahoo G.S., Hossain I., Wabaidur S.M., Ansari M.Z. (2023). Numerical investigation of toxic free perovskite solar cells for achieving high efficiency. Mater. Today Commun..

[28] Dong Z., Li W., Wang H., Jiang X., Liu H., Zhu L., Chen H. (2021). High-Temperature Perovskite Solar Cells. Solar RRL.

[29] Bhattarai S., Gogoi D., Sharma A., Das T.D. (2023). Performance enhancement by an embedded microlens array in perovskite solar cells. Indian J. Phys..

[30] Hima A., Lakhdar N., Benhaoua B., Saadoune A., Kemerchou I., Rogti F. (2019). An optimized perovskite solar cell designs for high conversion efficiency. Superlattices Microstruct..

